# Cholera Infantum

**Published:** 1849-11

**Authors:** 


THE NORTH-WESTERN

MEDICAL AND SURGICAL JOURNAL.

Vol. II.] NOVEMBER, 1849. [No. 4.

### Part I.—Original Communications.

### ARTICLE I.

*Cholera Infantum.* A paper read before the Central Wabash Medical Society.—By Dr. Ritchie.

In the earlier part of my practice, (in the year 1838–9,) I saw much more Cholera Infantum than I have seen since. In our town, (Newtown,) in 1838, it caused from 10 to 15 deaths. This was a greater mortality, I believe, than was ever produced in that place, in the same length of time, by any other disease, or all other diseases together. Though I believe I was not charged with being less fortunate in its treatment than my neighbors, yet, honestly, my experience was truly sad. The destroyer entered my own dwelling and carried away my first-born, my only son.

As the name imports, this disease is the Cholera of Infants; or as it is more commonly called, perhaps, "the Summer Complaint" of children. The common subjects of this disease are children under two years of age. To circumscribe it still more, we might say its subjects are children from four to eighteen months of age; or its period of attack is the period of first dentition. And the mothers are very apt to let it run on, and sometimes, indeed, too often, to a fatal termination, because, (as they say) "the child is cutting teeth."

*Symptoms.*—This disease very often approaches gradually in the form of diarrhœa, until, suddenly, from some cause, and often without any perceivable cause, the child is seized with violent vomiting and frequent cramps, and watery discharges from the bowels. Sometimes, indeed very often, these violent symptoms make their appearance without any previous diarrhœa, or any other premonition of an approaching attack. In the more urgent cases of this kind, the extremities are cold, eyes sunken, urgent thirst, and great prostration: and unless the vomiting and purging can be arrested, the child may die within the first 24 hours. In less severe cases, occasional vomiting and purging may continue for several days; then the vomiting may cease, and the purging continue. In such cases there is apt to be some febrile excitement present, especially in the afternoon, showing that this too, if it is not truly of an intermittent character, is modified very considerably by that prevailing tendency of almost all the diseases of this section of the country, to periodicity; the purging regularly being more troublesome during the earlier part of the day. As the disease advances, the child emaciates more or less rapidly; it becomes more fretful and irritable, till, in extreme cases, coma approaches, and it dies under the symptoms of Hydrocephalus.

The character of the discharges from the bowels varies much in different cases, and at different periods of the same case. Sometimes they are whitish and frothy, and at others mixed with undigested portions of food, then again they are slimy and mucous, and in many cases bloody. With the mucous and bloody discharges there is very apt to be great pain, during, and for some minutes previous to each evacuation, attended in some cases, with most awful bearing down and straining, showing extreme suffering. The green discharges, which are not uncommon, are by many considered billious. Some, I believe, have undertaken to account for them by supposing them to be bile mixed with some acid. By some experiments published by Dr. G. Bird, (for which I will refer you to Ranking's Abstract for 1845,) it was proven that there was no more bile in them than in common evacuations, so that with propriety, perhaps, they could not be called billious.—He supposed them to depend upon an an altered condition of the blood. What the change wrought in the blood is, I believe we are not informed. [I would merely suggest as an enquiry, whether it may not be the anæmic condition? I make this enquiry because I have seen those peculiar, light green evacuations where this was evidently the condition. Whether they may follow from any necessity, I am entirely unable to answer.]

In many cases of Cholera Infantum, the abdomen is tender upon pressure, and swollen, especially towards the termination of unfortunate cases. The appetite is variable, the tongue more commonly covered with a whitish crust: sometimes it is yellowish, and at other times red and smooth. The skin is mostly dry, and in many cases hot; the pulse is frequently somewhat excited; but I prefer to say but little about the peculiarities of the pulse, because it is very difficult in general to learn much about the pulse of a child.

*Cause.*—This disease has been ascribed to several causes. From the season of the year in which it is most common, (from May till September,) it is most probable that the heat of the summer has much to do with it. Whether this alone would produce it, we cannot tell. This with the vitiated atmosphere of a crowded city, favored by the irritation of dentition, unwholesome food, &c., no doubt can and does produce it in most of cases. Though it is most common and fatal in cities, yet we have enough of it in the country to try our patience and skill. Here, I have little doubt that excessive heat for a few days, followed suddenly by cool and damp weather, dentition, and improper diet, are the most fruitful sources of the disease. These causes may be favored by a miasmatic condition of the atmosphere, such as produces the common fevers of the country. Though Dr. Dunglison, I believe, supposes the miasm to be different from that which produces fever. I shall not attempt to dispute this point with such authority, for my impression is, if Dr. Dunglison is not the most learned, he is one of the most learned physicians now living; yet to me it would not be at all unreasonable to suppose that there may be some things take place up and down this river that even he might not know. But it is not necessary to dwell upon the causes of this disease, for it is not likely, if we knew them all, that our knowledge would prevent it in very many cases.

*Pathology.*—As to the Pathology of this disease, I can have but little to say from experience; for honesty always being the best policy, I am ready to admit at once, that I never witnessed a "*post mortem*" in such a case. Where death occurs very early, we should not expect to find much, if any evidence of inflammation. Where the disease is protracted, from the symptoms, and from the reports of those who have made, after death, examinations of such cases, we should expect to find increased redness, at least, in different portions of the alimentary canal. In some cases, contraction of a portion of the intestines is met with. In most of cases it is said there is enlargement of the muciparous glands; and in some cases ulcerations. The liver is said to be congested, enlarged, and in some cases light colored. Effusion into the ventricles, and upon the surface of the brain, is frequently found. These, perhaps, would constitute the most common pathological conditions. And I would here remark, that physicians should more diligently seek opportunities to make post mortem examinations in this, and other diseases, inasmuch as without them we must often be found using our means in the dark.

*The Prognosis* in this disease must depend very much upon the case you have to treat; and then if you have, seemingly, the mildest case, if the child have any scrofulous taint; or if it have a great many teeth to cut; or, if it has been lately weaned, or have to suck the milk of a pregnant mother, you had better be cautious in making an early, favorable prognosis. Though it is said to have produced in the city of Philadelphia, "from 1825 to 183[illegible] inclusive, 3,352 deaths, being near 10 per cent. of all the deaths of children under 5 years of age, and near 5 per cent. of the entire mortality of the city in that period," yet it is nevertheless true, that by far the greater proportion of cases recover, and if judicious means are used early in the disease, comparatively few will die.

*Treatment.*—If you are consulted early in the disease, when it is in the form of moderate diarrhœa, you will first, if you can ascertain the cause, remove it; if the gums are swollen, cut down freely upon the approaching teeth. A flannel bandage should be worn around the abdomen, and the feet and arms kept warm.

A common practice, and in many cases it seems a very good one, is to give a portion of Castor Oil with from one to five drops of Tinc. Opii., according to the age and habit of the patient. In most of cases perhaps it would be better to give a small portion of Cal. and Rhubarb, to be followed, if need be, by a Dovers Powd. This, in many cases, arrests the disease at once. Cal., 1-4 to 1-6 of a gr., and Ipec. 1-4 gr. every hour and a half to two hours has been a common and leading prescription in our section, and in many cases a very satisfactory one; yet I am inclined to believe it has been overrated and too much relied upon by many physicians. My impression now is, that I too, in times past, have had too much confidence in it. The idea was, that we must have the deranged secretions of the liver, skin and alimentary canal corrected, (all very well, certainly,) and these two articles were to do the work; and they would seem to look in that direction, to be sure; but unfortunately, in many cases not sufficiently. To answer these ends more generally and efficiently, I now have much more confidence in the warm bath, employed daily, and the following formula which I got from Dr. Condies' work on Children, which in my practice has given very general satisfaction, viz: Cal. grs. iii; Cret. ppt. grs. xxxvi; Acet. Plum. grs. xii; Ipec. grs. iii, divide into 12 parts, one to be taken every 3 hours. This generally changes the character of the discharges in a short time, and promptly arrests the disease.

To arrest the vomiting, which is frequently a very troublesome and urgent symptom, I have found minute portions of Cal., 1-4 to 1-6 of a gr. thrown back upon the tongue every half hour, to arrest it in a short time. Should this, with a large sinapism to the epigastrium not succeed, I would give a few drops of Spts. Turp., or a few drops of Sul. Ether, holding in solution some Gum Camph: Ether, 1 oz.; Camph., 1 ʒ. Where the common means of arresting vomiting fails, it is advised to use a solution of the Acet. Plum. I have tried it in only two cases, and in one of these it too failed, and the child died—the only case, I believe, I ever saw terminate fatally before the vomiting was arrested. It may be necessary to apply a blister to the stomach, and sinapisms to the legs. In blistering children, however, it should be kept in mind that the plaster should be replaced by a poultice whenever the skin is well reddened, or after from two to three hours. When the vomiting is arrested, the powders of Cal., Chalk, &c., above mentioned, should be resorted to and continued till the purging is subdued, leaving out the lead whenever the violence of the disease i[illegible]vercome; or when the frequent watery discharges disappear. To arrest the vomiting and purging too, in a case where both were very copious, frequent and prostrating, the discharges being thrown some feet from the anus,

I gave Tinc. Opii. *per orem*, and also by injection, till I had from 20 to 25 drops in the little fellow, (a child of 9 to 10 months of age,) at one time. It succeeded entirely and when the child recovered from the prostration produced by the disease, it was well. Some might think this hazardous---I tho't the disease more so. Some physicians, I apprehend, are too fearful of Opium in diseases of children. Dr. Dixon, now of the New York University, gives Camph., Tinc. of Opii. and Carb. of Potash, to arrest the vomiting in this disease; and I have no doubt it would be good for the purging also in most cases, for signs of acidity are very apt to be present in these cases, and every physician knows that a great many diarrhœas may be cured by alkalies alone.

When there is much pain in the bowels, with slimy and bloody discharges, injections of Tinc. Opii. with Mucilage of Slippery Elm or thin Starch, is a means of great relief, and in some cases it would seem could not be dispensed with. They should be given as occasion requires. Where the abdomen is swollen and tender, attended with much griping and bearing down during the evacuations, the Spts. Turp., with Mucilage of Gum Arabic or Elm and Calc. Mag. is a mixture of great value---better if a little Tinc. Opii. or Tinc. Camph. be added. In persistent cases, where there is not much evidence of local inflammation present, astringents, as Kino and Catechu may be used. In such cases too, a blister over the abdomen may do good by its derivative action. Poultices to the abdomen are of great value in the treatment of this disease, and still more valuable if made irritating by pepper or mustard. These, as a general rule, I prefer to blisters, as children, owing to some peculiarity of system, do not bear blistering so well as adults; however, where there is evidence of inflammation of the brain, they must be applied to the temples, behind the ears, or to the nape of the neck, and kept sore while it continues, by some stimulating ointment. Cold water should also be applied to the head in such cases. A bladd[illegible]

partly filled with cold water and laid to the head, is perhaps the best mode of applying it. In a great many cases, where a number of teeth are to be cut, and the case becomes lingering, wearing the child away, rather by its continuance than its violence, the best thing that can be done. is to keep the diarrhœa in check by the administration of Dover's Powders as occasion requires. In cases of much emaciation and debility, tonics must be used. I have little doubt that many of the cases treated as inflammation of the brain, connected with this disease, are cases of drowsiness from mere debility, and require tonics. Quinine and Iron may be used. Quinine is especially called for in cases of an intermittent character. Where there is evidence of anæmia we would use the Iron. But in most of cases, the one I have been most pleased with, is the powdered Columba. This will give very considerable support, and agree well with the irritated bowels. After all that may be said, the Dover's Powders, the warm bath, and the Calomel, Chalk and Lead powders above mentioned, are the main reliance in most of cases. The diet should be fluid and unirritating, and the quantity small at a time, and not taken too frequently. The child, in pleasant weather, should be carried on horse-back, or in a carriage, and removed to the country if in a city, or by all means to a healthy situation, if possible.

I cannot close these remarks without insisting upon the necesity of the positive prohibition of solid indigestible articles of diet. Fruits of all kinds should be forbidden, as one mouthful continues a diarrhœa for days or weeks in many cases, and too often no doubt, gives a fatal termination to the disease. One case I will mention. Some years ago a respectable physician of my acquaintance was attending a child laboring under Cholera Infantum. The child was evidently convalescent for a few days, when the doctor happening to pass the house, called to see how the little patient was getting along, and found it sitting up in the cradle feeding upon cucumbers from the kind hand of its mother. The doctor remonstrated with her upon the impropriety of feeding the child on such diet, and faithful to his conviction, hinted the probability that death, even, might be the consequence of such indiscretion.--- "Ah! doctor," she religiously replied, continuing the feed at the same time, "I don't believe it will ever die till its time comes." The result was, the disease very soon returned in great violence, and by the next morning 'its time had come.'

